# Acutely Symptomatic Hypoperfusion Through an Occluded Subclavian to Internal Carotid Artery Bypass Graft: Salvage Mechanical Thrombectomy and Graft Revascularization

**DOI:** 10.7759/cureus.20881

**Published:** 2022-01-02

**Authors:** Jose Marino Granados, Anna Luisa Kuhn, Ajit S Puri, Francesco Massari, Jasmeet Singh

**Affiliations:** 1 Department of Neurology, University of Maryland Medical Center, Baltimore, USA; 2 Division of Neurointerventional Radiology, Department of Radiology, University of Massachusetts, Worcester, USA

**Keywords:** carotid artery, stent, stroke, mechanical thrombectomy (mt), bypass graft

## Abstract

This middle-aged patient had undergone surgical placement of a left subclavian to internal carotid artery bypass graft five years ago for treatment of symptomatic, chronic, bilateral carotid occlusions. The patient was neurologically intact after the procedure and until the day of presentation with symptoms of an acute left anterior circulation stroke. Initial workup confirmed acute occlusion of the graft as the cause of the patient’s symptoms. Endovascular recanalization of the bypass graft in the setting of chronic bilateral carotid occlusions was a technical and conceptual challenge. Simultaneous radial and femoral vascular access allowed for direct recanalization of the graft (through thrombectomy, angioplasty, and stent placement) with intraoperative patency surveillance of the circle of Willis via the posterior circulation. Most of the neurological deficits improved, and the patient was discharged to rehabilitation close to neurologic baseline.

## Introduction

Bypass surgeries of the brachiocephalic arteries are less prevalent today than they were in the past. Today, patients can be treated successfully using minimally invasive endovascular devices. However, the presentation of an acute stroke secondary to a bypass graft occlusion can lead to enormous diagnostic and therapeutic difficulties. Our case report offers a technical description of how to recanalize the graft and concomitantly monitor for (and treat) emboli traveling from the distal end of the graft to the intracranial circulation.

## Technical report

**Figure 1:**
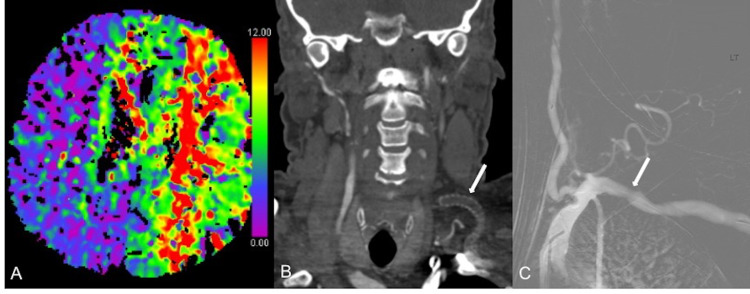
Stroke Imaging CT perfusion demonstrates a large area of ischemic penumbra involving the entire left anterior circulation and right anterior cerebral artery territory (A). CT angiogram of head and neck (coronal plane) shows complete occlusion of the left subclavian to ICA bypass graft (B, arrow). Angiographic image confirms complete occlusion of the bypass graft (C, arrow).

The patient was a middle-aged, right-handed man, with a history of hyperlipidemia, type 2 diabetes mellitus, hypertension, atrial fibrillation, a prior right cerebellar stroke without sequela, and known bilateral carotid occlusions. The patient was also status post placement of a left subclavian to left internal carotid artery (ICA) bypass graft approximately five years ago for treatment of symptomatic, bilateral carotid occlusions. Of note, the patient was noncompliant with several medications including antiplatelet therapy, anticoagulation, antihypertensives, and insulin. Despite the prior cerebellar stroke and bilateral carotid occlusions, the patient’s modified Rankin score was 0. Upon waking up on the morning of presentation, the patient noticed right upper extremity weakness and pain. A blood pressure check at home showed a systolic blood pressure (SBP) of 190 mmHg. The patient then decided to take an unknown amount of metoprolol. As the blood pressure decreased, dysarthria, aphasia, right facial droop, right-sided neglect, and right upper and lower extremity weakness started to develop. Though the symptoms fluctuated, blood pressure enhancement failed to achieve a return to the patient’s neurologic baseline, and at its worse, the National Institute of Health Stroke Scale (NIHSS) was 17. Initial workup included an unremarkable noncontrast head CT, CT perfusion imaging which showed a 3 cm x 3 cm infarct core in the posterior left temporal lobe and a large ischemic penumbra involving the entire left anterior circulation as well as the right anterior cerebral artery territory (Figure 1A). CT angiogram of the head and neck demonstrated occlusion of the left subclavian to left ICA graft, occlusion of the right petrous and cavernous ICA, patency of all other intracranial vessels (with a hypoplastic right A1), and moderate stenosis at the origin of the right vertebral artery with otherwise patent vertebrobasilar system (Figure 1B). The approach to the endovascular treatment detailed below consisted of four consecutive stages: 1) initial diagnostic cerebral angiogram, 2) guidewire and catheter exploration of the occluded bypass graft, 3) mechanical thrombectomy of the left M1 occlusion secondary to the instrumentation of the occluded bypass graft, and 4) revascularization of the bypass graft. The procedure was performed under general anesthesia. Dual arterial access was obtained with 6-French arterial sheaths in the left radial and right common femoral arteries. Tight blood pressure control was maintained (140-180 mmHg systolic). Anesthesia team was able to use the arterial pressure recordings from a transducer connected to the side port of the groin sheath. From the right groin approach, a 4-French Berenstein catheter (Cordis, Miami Lakes, FL, USA) was navigated into the left subclavian artery. The left vertebral artery was selected with the catheter. Cerebral angiogram through the left vertebral artery showed filling of the entire intracranial vasculature via the posterior circulation with filling of the anterior circulation via the posterior communicating arteries. The angiogram of the left subclavian artery did not show any opacification of the bypass graft (Figure 1C).

Another 4-French Berenstein catheter was navigated into the subclavian artery from the left radial approach due to the more favorable angle for graft catheterization, as per the CT angiogram. The graft itself was radiolucent and not visible on fluoroscopy. The expected take-off of the graft was gently, blindly probed with a 0.035-inch Glidewire (Terumo, Fremont, CA, USA). The wire could be advanced into the graft against some minor resistance. The Berenstein catheter could then be navigated into the left cervical ICA across the graft. Intraluminal position was confirmed with a gentle contrast injection.

**Figure 2:**
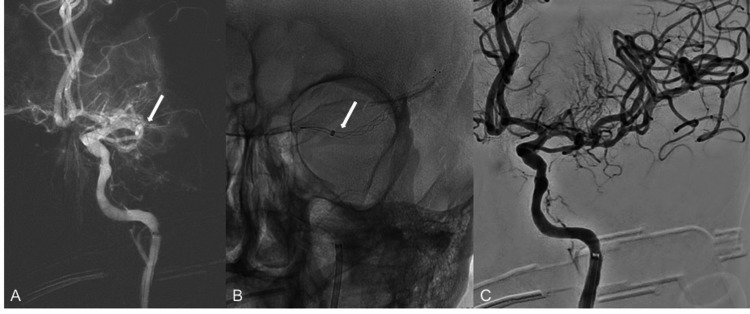
Salvage Mechanical Thrombectomy Despite initial patency, after manipulation of the graft’s thrombus, a distal embolus dislodges and occludes the left middle cerebral artery (MCA) (A, arrow). Triaxial technique is used for thrombectomy of the left MCA occlusion with deployment of a stent-retriever and aspiration catheter (B, arrow). Contrast injection from the left internal carotid artery (ICA) immediately after thrombectomy shows recanalization of the MCA (C) with slow flow in a few distal cortical vessels (Thrombolysis in Cerebral Infarction (TICI) 2c).

Follow-up angiography revealed an occlusion of the left M1 segment (Figure 2A). This was likely secondary to the wire and catheter navigation through the thrombus filled bypass graft. The diagnostic catheter was exchanged for a 6-French Benchmark catheter (Penumbra, Alameda, CA, USA) over a Glidewire Advantage wire (Terumo). Using a triaxial technique [1], a 5-French Sofia aspiration catheter (Microvention, Aliso Viejo, CA, USA) was navigated over a Trevo ProVUE 21 microcatheter (Stryker, Fremont, CA, USA) and a Synchro 0.014″ microwire (Stryker) to reach the superior division of the left middle cerebral artery (MCA). Once there, a Trevo 6 mm x 40 mm thrombectomy device (Stryker) was deployed from the superior division M2 segment into the left M1 (Figure 2B). Mechanical aspiration thrombectomy was performed in a standard fashion. Follow-up angiogram evidenced patency of the left MCA (Figure 2C) with slow flow in a few distal cortical vessels (Thrombolysis in Cerebral Infarction (TICI) 2c).

**Figure 3:**
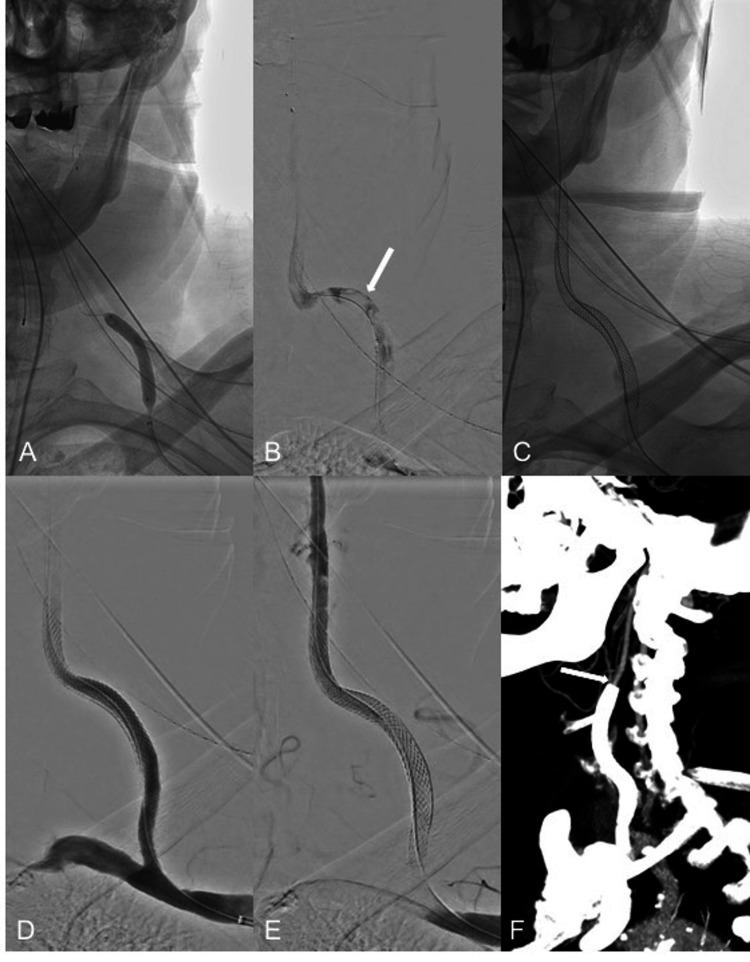
Bypass Graft Recanalization Multiple overlapping angioplasties are performed along the entire length of the bypass graft (A). Persistence of high clot burden throughout the entire graft is seen (B, arrow). Placement of three overlapping self-expanding carotid stents including a balloon-mounted stent at the site of the proximal anastomosis of the graft (C). Final angiogram images show recanalization of the bypass graft (D and E). Ten-month follow-up CT angiogram shows a patent bypass graft (F, arrow).

After flow through the MCA was restored, a 5-mm Spider embolic protection device (Medtronic, Irvine, CA, USA) was deployed at the level of the left cervical ICA. Multiple overlapping angioplasties of the graft were then performed using a 5 mm x 20 mm over-the-wire balloon (Figure 3A). There was persistent angiographic evidence of extensive clot burden throughout the graft (Figure 3B). Multiple attempts at mechanical aspiration thrombectomy were also performed leading to improvement of the caliber of the graft; however, persistent recoil of the graft kept resulting in reocclusion of the lumen.

At that point, it was decided to trap the residual clot in the graft using closed-cell stents. Three overlapping self-expandable Carotid Wallstents (8 mm; Boston Scientific, Santa Clara, CA, USA) were placed along the entire length of the graft (Figure 3C). A balloon-mounted stent was deployed at the site of the proximal anastomosis with the native subclavian artery for anchoring purposes. Follow-up angiogram showed patency of the bypass graft (Figures 3D, 3E). Regarding the intracranial recanalization, there was complete opacification of the distal left ICA and MCA. A small embolus was seen in a distal anterior cerebral artery branch.

The patient’s initial NIHSS was 17 (for dysarthria, aphasia, right facial droop, right-sided neglect, and right upper and lower extremity weakness). At the time of discharge, the NIHSS had declined to 3 (right facial droop, mild mixed aphasia, and mild disorientation). MRI was not obtained given the presence of metallic hardware that precluded the patient from undergoing the study, but follow-up noncontrast head CT only showed evolution of a left caudate stroke. The patient was discharged to a rehabilitation facility on dual antiplatelet therapy with aspirin 81 mg (lifelong) and clopidogrel 75 mg (for three months), in addition to warfarin for his atrial fibrillation. A clinical follow-up at three months showed that the patient has fully recovered. A follow-up CT angiogram 10 months after the procedure showed continued patency of the bypass graft (Figure 3F).

## Discussion

Presentation of severe atherosclerotic disease of the carotid arteries leading to bilateral obstruction can vary from incidental asymptomatic findings to fulminant ischemic strokes, depending on the degree of collateral circulation and the chronicity of the atherosclerotic lesions. Prior to the age of endovascular therapy, bypass techniques of the brachiocephalic arteries were a standard therapeutic approach for management of symptomatic occlusions [2]. As these surgical options became less prevalent, the available data looking into the rate of occlusion and the safest approaches for recanalization also became scarce. There are limited data regarding the incidence of reocclusion of subclavian-carotid bypass grafts; earlier case series suggested that such events are unlikely [3]. A retrospective study of 153 patients [4] showed that endovascular treatment with angioplasty and stenting of obstructed brachiocephalic bypass grafts was a viable alternative for management.

In this case, the combination of multiple vascular risk factors and medication noncompliance led to an atypical anatomical presentation of an acute stroke. The symptomatology exhibited corresponded to hypoperfusion of the left anterior cerebral circulation on someone with known chronic bilateral carotid occlusions who had already undergone a bypass graft from the left subclavian artery to the left internal carotid artery, distal to the native obstruction. This hypoperfusion was initially confirmed by angiographic findings that showed nonopacification of the bypass graft and by perfusion imaging showing a small ischemic core with a large ischemic penumbra that correlated with the patient’s symptoms. Treatment of acute ischemic stroke in the setting of an altered anatomy secondary to the placement of a surgical bypass can be challenging, especially when the graft is the culprit “vessel” of the patient’s symptoms. Recanalization of the occluded vessel via mechanical thrombectomy is the gold standard for treatment of ischemic strokes; however, the specific approach and surgical planning in our case must take into consideration not only the occlusion of the graft but also the prior and permanent occlusions of the native vessels. Endovascular recanalization of an acutely occluded subclavian-carotid graft for the treatment of an acute ischemic stroke has not been described previously. Given that any instrumentation of the thrombotic segment could lead to embolization from the distal end of the occluded graft to the anterior circulation, preprocedural planning was of paramount importance and it prioritized a dual access model that allowed for real-time evaluation of the intracranial circulation via angiograms from the vertebrobasilar system while concomitantly using the left radial access to directly instrument the occluded bypass graft. Initial angiographic imaging via the posterior circulation showed initial patency throughout the circle of Willis through collateral flow from the posterior communicating arteries. As foreseen, initial manipulation of the occluded graft led to an embolus that blocked flow through the MCA. Fortunately, this did not lead to further complications as prompt mechanical thrombectomy was successfully performed. Further embolic events were prevented by deployment of a distal protection device within the cervical ICA. The high clot burden within the graft made direct aspiration therapy insufficient for recanalization. Deployment of overlapping stents not only helped with “trapping” of the remaining clot but also conferred sustained patency of the graft and optimal flow to the anterior circulation.

## Conclusions

Recanalization of a subclavian to carotid bypass graft via direct aspiration, angioplasty, and endovascular stenting should be considered as a feasible alternative for patients with a clinical presentation of an acute ischemic stroke.
